# Dealing with a soft tissue lesion that is scheduled for CT-guided biopsy and that has decreased in size on preprocedural planning CT

**DOI:** 10.1259/bjrcr.20190071

**Published:** 2020-02-12

**Authors:** Derya Yakar, Thomas C Kwee

**Affiliations:** 1 Medical Imaging Centre, Department of Radiology, University of Groningen, University Medical Centre Groningen, Groningen, The Netherlands

## Abstract

On planning CT before CT-guided biopsy, the target lesion may have decreased in size compared to previous imaging. Radiologists frequently face the dilemma of whether to biopsy these shrinking lesions or not. There is currently a lack of literature on how often such a situation is encountered in clinical practice, how it is dealt with, and if the perceived lesion size reduction always implies benignancy. This information would be valuable to develop evidence-based strategies for this specific clinical situation. We aimed to determine the frequency, radiologist’s management, and nature of lesions with size reduction on prebiopsy planning CT. In this retrospective study, we found that the incidence of lesions with size reduction on prebiopsy planning CT was 1.00% (11/1103). Biopsy was refrained from in most cases (9/11). Eight lesions proved to be benign, one malignant, one harboured both benign and malignant pathology, and one lesion remained of unclear nature. Soft tissue lesions with size reduction on prebiopsy planning CT are encountered infrequently and are usually not biopsied. Although most of these lesions are benign, lesion size reduction does not exclude malignancy. Therefore, clinical and imaging follow-up should be considered mandatory when biopsy is cancelled.

## Introduction

CT-guided percutaneous needle biopsy is a well-established technique to acquire tissue for pathological examination.^1^ CT allows for visualisation of the location of the target lesion and its surroundings as well as stepwise controlling of instrumentation necessary for biopsy. Therefore, it is generally a safe and efficient procedure.^1,2^ In patients who are referred for CT-guided biopsy, the main purpose of the procedure is commonly to rule in or rule out a malignant diagnosis.^3^ In some instances, the target lesion may have decreased in size on preprocedural planning CT compared to previous imaging examinations, as determined by the attending radiologist. Radiologists frequently face the dilemma of whether to biopsy a shrinking lesion or not. While decreasing lesion size may be attributed to benignancy and technical difficulty with biopsy, time allocation and satisfaction of tissue acquisition for a gold standard diagnosis may encourage the radiologist to obtain the biopsy. However, there is currently a lack of literature on how often such a situation is encountered in clinical practice, how it is dealt with, and if the perceived lesion size reduction always implies benignancy. This information would be valuable to develop evidence-based strategies for this specific clinical situation. Therefore, this study aims at determining the frequency, radiologist’s management, and nature of lesions that decrease in size on prebiopsy planning CT.

## Methods and materials

This retrospective study was approved by the local institutional review board (registration number 201800105) and the need for informed consent was waived. All patients who participated in CT-guided core needle biopsy of a soft tissue lesion at our academic tertiary care centre between January 2005 and December 2017 were eligible for inclusion. CT-guided cytological aspirations and CT-guided biopsies of bone lesions and (suspected) spondylodiscitis were excluded. A total of 1103 consecutive soft tissue biopsies were performed in 1027 unique patients (66 patients underwent biopsy on two different occasions, and five patients underwent biopsy on three different occasions) during this 13-year period. These 1027 patients consisted of 590 males and 437 females, with a mean age ±SD of 60.0 ± 15.8 years (range: 1–91 years). Biopsies were (scheduled to be) performed in the lung (*n* = 567), abdomen (*n* = 269), pelvis (*n* = 78), mediastinum (*n* = 57), paraspinal region (*n* = 48), chest wall (*n* = 35), extremities (*n* = 30), head-neck (*n* = 10), parasacral region (*n* = 5), heart-pericard (*n* = 2), and abdominal wall (*n* = 2). All procedures were performed with use of a 16- or 64-slice CT system (Siemens SOMATOM Sensation 16 or Siemens SOMATOM Definition 64). All 1103 radiology reports were reviewed to determine if the reporting radiologist mentioned a decrease in lesion size, and how it was subsequently handled in terms of non-execution of the biopsy. Axial long-axis diameters of lesions that were reported to have decreased in size were retrospectively measured by a radiologist (<blinded>), if possible. Malignant or benign nature of these lesions was determined based on available pathology reports and clinical and imaging follow-up.

## Results

**Figure 1.**
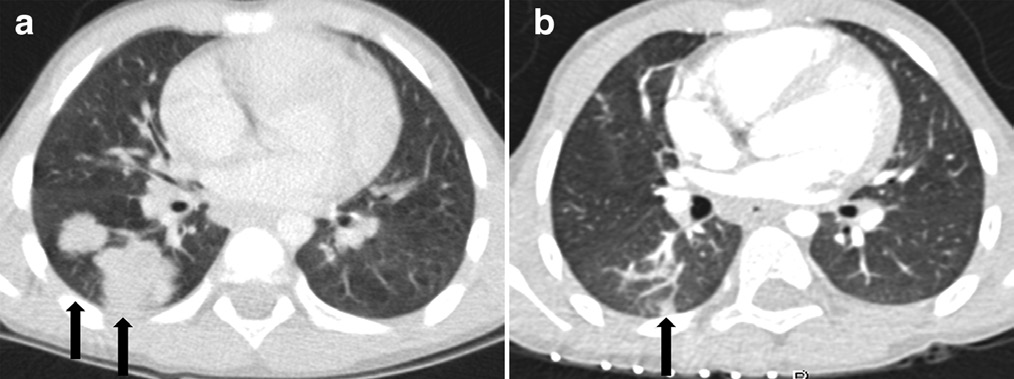
A 7-year-old boy was admitted to the hospital with fever (patient no. two in Table 1). Conventional chest X-ray (not shown) and CT (1A) showed multiple lung lesions (black arrows). On the prebiopsy planning CT (1B), the lesions had either disappeared or considerably decreased in size (black arrow). No biopsy was performed. The patient received antibiotics and completely recovered at 3-month follow-up. The lung lesions were probably attributable to infection.

**Figure 2.**
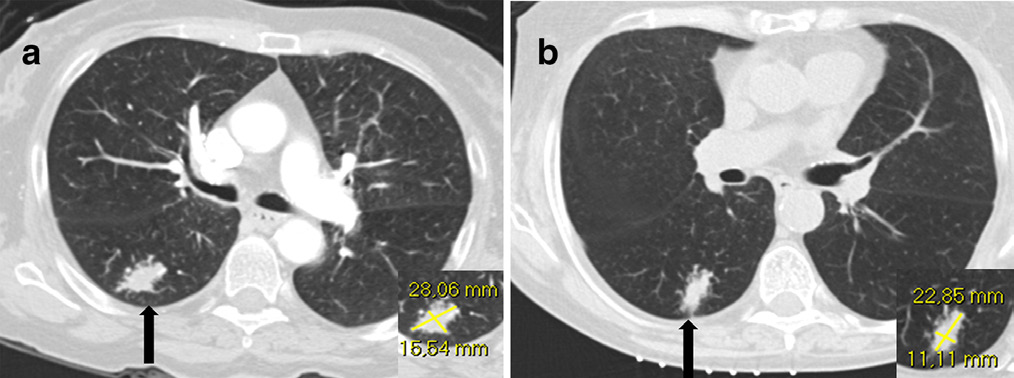
A 60-year-old female was admitted to the hospital because of headache (patient no. 11 in Table 1). CT and MRI of the brain (not shown) demonstrated multiple cerebellar lesions suspicious for metastases. Subsequently, a CT scan of the thorax and abdomen was performed which revealed a lesion in the lung (1A) (black arrow) of 28 mm long-axis diameter. On prebiopsy planning CT, this lesion had decreased in size to 23 mm (1B) (black arrow). The attending radiologist decided to proceed with biopsy. Subsequent pathological examination revealed a large-cell neuroendocrine carcinoma.

**Figure 3.**
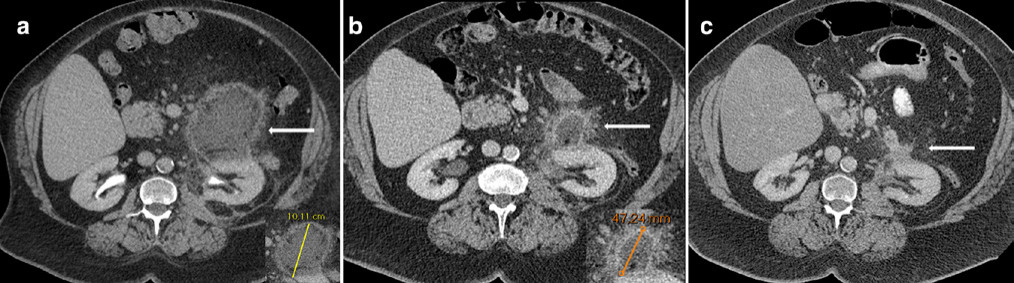
A 67-year-old female was referred to the urologist because of a history of repeated urinary tract infections and recent episodes of abdominal pain. The ordered CT scan (3A) showed a large retroperitoneal lesion (white arrow) measuring 10.1 cm in long-axis diameter (zoomed inset). The patient’s family history revealed a mother who had died from an “adrenal tumour” and a brother who had died from a retroperitoneal sarcoma. Based on the CT findings and the patient’s family history, a retroperitoneal sarcoma could not be excluded. On the prebiopsy CT scan (3B), which was performed 81 days later, the lesion had substantially decreased in size, measuring 4.7 cm (white arrow and zoomed inset). The attending radiologist decided to abort the biopsy. After a 7-month clinical and CT (3C) follow-up that demonstrated a further size decrease of the lesion (white arrow), it was concluded that the lesion probably represented a retroperitoneal haematoma (due to acetylsalicylic acid and clopidogrel use because of a history of hypertension and acute coronary syndrome) and no further follow-up was deemed necessary.

**Figure 4.**
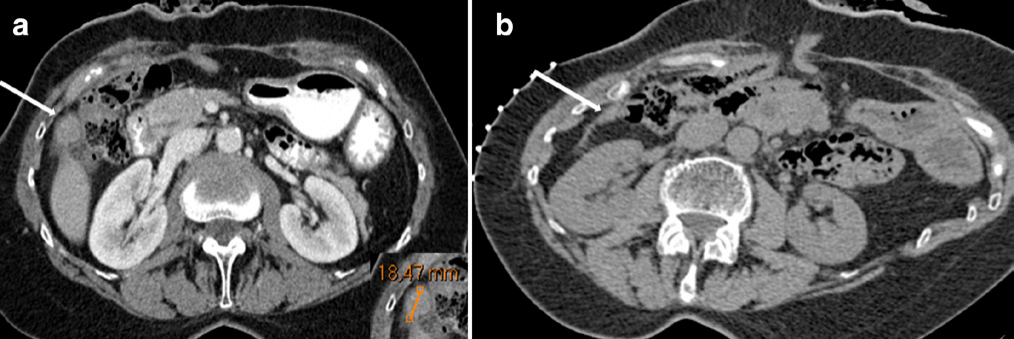
A 69-year-old female with a previous history of rectal cancer and a solitary liver metastasis (for which she was surgically treated) underwent routine follow-up CT (4A) on which a lesion (white arrow) measuring 1.8 cm in long-axis diameter (zoomed inset) was seen. On the prebiopsy CT scan (4B), which was performed 46 days later, the lesion (white arrow) had decreased in size and was not reliably measurable anymore. However, the attending radiologist decided to perform the biopsy anyway. Histopathology revealed muscle and fat tissue, and no malignant, inflammatory, or other lesional tissue. A 49-month clinical and CT follow-up (demonstrating complete resolution of the lesion) confirmed the benign nature of this lesion.

11 out of 1103 soft tissue lesions that were about to undergo CT-guided biopsy had decreased in size on preprocedural planning CT compared to previous imaging according to the radiology report, which corresponds to a frequency of 1.00% (95% CI: 0.56–1.78%). These 11 soft tissue lesions were present in 11 unique patients (six males and five females, with a median age of 60 years [range: 7–81 years]). Retrospective review confirmed the decrease in lesion size in all 11 cases. In two cases, the lesions were not even visible anymore on prebiopsy planning CT. Nine of the lesions were located in the lung, one was located in the retroperitoneum, and one was located perihepatic. Almost all of the lesions were suspicious of malignancy or metastasis at the time of (scheduled) biopsy. In nine cases, the attending radiologist decided to not perform the biopsy after consulting the referring physician. In two cases, the radiologist proceeded to perform the biopsy, without consulting the referring physician. According to available pathology reports in three patients, and clinical and imaging follow-up available in seven patients, eight lesions proved to be benign (the majority due to infection), one was malignant (pathologically proven pulmonary large-cell neuroendocrine carcinoma based on the CT-guided biopsy), one harboured both benign and malignant pathology (pathologically proven infection and low-grade lymphoplasmacytic lymphoma based on autopsy), and the nature of one lesion remained unclear. Further details of all cases are displayed in Table 1. CT-scans of four cases are shown in Figures 1–4.

**Table 1.** Overview of 11 patients who had a lesion that had decreased in size on prebiopsy planning CT

Patient no.	Age	Gender	Location lesion	Working diagnosis before biopsy	Lesion size on previous imaging^a^	Lesion size on planning CT^a^	Days between previous imaging and planning CT	Biopsy done?	Final diagnosis	Reference standard
1	62	F	Abdomen (retroperitoneal)	Malignancy	10.1 cm^b^	4.7 cm^b^	81	No	Benign (probably haematoma)	7-month clinical and CT follow-up
2	7	M	Lung	Metastasis, infection	Num^b^	Num^b^	10	No	Benign (probably infection)	3-month clinical and radiographical follow-up
3	43	F	Lung	Malignancy, infection, inflammation	3.1 cm^b^	1.6 cm^b^	55	No	Benign (probably infection)	8-month clinical and FDG-PET/CT follow-up
4	52	M	Lung	Metastasis, infection	4.0 cm^c^	2.6^c^	57	No	Benign (probably infection)	4-month clinical and FDG-PET/CT follow-up
5	57	F	Lung	Malignancy, infection	3.9 cm^c^	NV^b^	59	No	Benign (probably infection)	7-month clinical and CT follow-up
6	81	M	Lung	Malignancy, infection	NRM^c^	NV^b^	20	No	Benign and malignant (infection and low-grade lymphoplasmacytic lymphoma)	Autopsy
7	38	M	Lung	Infection	Num^c^	Num^c^	8	No	Benign (probably infection)	6-month clinical and FDG-PET/CT follow-up
8	64	M	Lung	Malignancy	NRM^a^	NRM^c^	14	No	Unclear	Not available^d^
9	79	M	Lung	Malignancy, infection	7.8 cm^b^	49^c^	42	No	Benign (probably infection)	1-month clinical and radiographical follow-up
10	69	F	Abdomen (perihepatic)	Metastasis	1.8 cm^b^	NRM^c^	46	Yes	Benign	Biopsy (no malignancy) and 49-month clinical and CT follow-up
11	60	F	Lung	Malignancy	2.8 cm^b^	2.3^c^	19	Yes	Malignant (large-cell neuroendocrine carcinoma)	Biopsy

Num: not a clear single target lesion, but numerous lung lesions that decreased in size

NV: not visible anymore

NRM: not reliably measurable

a Axial long-axis diameter

b On contrast-enhanced CT

c On unenhanced CT

d Ground-glass lesion decreased in size, but its nature could not be determined anymore on follow-up imaging due to the administration of chemotherapy for lung adenocarcinoma at another location

## Discussion

Currently, there are no published protocols or guidelines advising on whether to biopsy a lesion that has decreased in size on prebiopsy planning CT. First, our results demonstrate that lesion size reduction on prebiopsy planning CT is relatively uncommon. Therefore, most radiologists will lack clinical experience in how to appropriately deal with such lesions. Nevertheless, in the majority of such cases, the biopsy is cancelled by the radiologist. This is particularly justifiable for lesions that have decreased in size to such an extent that biopsy is technically not possible anymore. In our series, the decision to not biopsy was always jointly made by the attending radiologist and the referring physician. All patients invariably underwent subsequent clinical and imaging follow-up. Although the majority of lesions that decreased in size proved to be benign, clinical and imaging follow-up should be considered mandatory to confirm benignancy when biopsy is cancelled, because lesion size reduction does not exclude malignancy. In our series, two lesions harboured malignancy on pathological examination. The first case was a patient with a pleural lesion that was not visible anymore on prebiopsy planning CT and that represented a combination of infection and low-grade lymphoplasmacytic lymphoma on autopsy. The second case was a patient with a lung lesion that decreased in size and that proved to be a large-cell neuroendocrine carcinoma on pathological examination of the biopsy specimen. We speculate that lesion size reduction in these two cases was due to a decrease of an accompanying infectious component.

This study had some limitations. First, although a total of 1103 CT-guided soft tissue biopsy procedures were reviewed over a 13 year period, the number of lesions that had decreased in size on preprocedural planning CT was still very low. Therefore, no further analyses could be done to investigate which clinical and imaging variables are associated with benignancy or malignancy in this small subset of lesions that experienced size reduction. Second, although lesion size reduction was confirmed by retrospective review, it was initially determined based on the dictated radiology report. Furthermore, some lesions were either not reliably measurable or had completely become invisible. Therefore, no clear cut-off value can be given as to what constitutes a lesion size reduction. Third, pathological confirmation was available in only 2 of 11 lesions. Nevertheless, all other lesions (except one whose nature remains unclear) could be classified with use of clinical and imaging follow-up.

In conclusion, the incidence of lesions that decrease in size on prebiopsy planning CT is very low. The radiologist usually refrains from biopsying these lesions. Although most of these lesions are benign, lesion size reduction does not exclude malignancy. Therefore, clinical and imaging follow-up should be considered mandatory when biopsy cancellation is opted for.

## Learning points

- The incidence of lesions that decrease in size on prebiopsy planning CT is very low.
- The radiologist usually refrains from biopsying these lesions.
- Although most of these lesions are benign, lesion size reduction does not exclude malignancy. Therefore, clinical and imaging follow-up should be considered mandatory when biopsy cancellation is opted for.

## References

[1] Haaga JR Interventional CT: 30 years’ experience. Eur Radiol Suppl 2005; 15(S4): d116–20. doi: 10.1007/s10406-005-0130-9 16479660

[2] Nattenmüller J, Filsinger M, Bryant M, Stiller W, Radeleff B, Grenacher L, et al Complications in CT-guided procedures: do we really need postinterventional CT control scans? Cardiovasc Intervent Radiol 2014; 37: 241–6. doi: 10.1007/s00270-013-0673-4 23778886

[3] Gupta S, Wallace MJ, Cardella JF, et al Society of interventional radiology standards of practice Committee. quality improvement guidelines for percutaneous needle biopsy. J Vasc Interv Radiol 2010; 21: 969–75. 20304676 10.1016/j.jvir.2010.01.011

